# Nivolumab-Induced Fasciitis Mimicking Scleroderma: A Diagnostic Challenge

**DOI:** 10.7759/cureus.90484

**Published:** 2025-08-19

**Authors:** Rida Jannat, Michael Kshatri, Rupak Thapa

**Affiliations:** 1 Rheumatology, Wake Forest Baptist Medical Center, Winston-Salem, USA; 2 Internal Medicine, Wake Forest Baptist Medical Center, Winston-Salem, USA; 3 Rheumatology, Atrium Health Wake Forest Baptist Health, Winston-Salem, USA

**Keywords:** eosinophilic fasciitis, immune check point inhibitors, iraes, nivolumab-related adverse events, scleroderma mimic

## Abstract

A 74-year-old male on nivolumab for desmoplastic melanoma developed progressive skin tightening, woody induration, and restricted mobility, initially suggestive of systemic sclerosis. He lacked classic scleroderma features (e.g., Raynaud’s, digital ulcers) but had eosinophilia (1100 cells/µL) and antinuclear antibody (ANA)/ ribonucleoprotein (RNP) positivity. MRI confirmed eosinophilic fasciitis (EF), prompting discontinuation of nivolumab and initiation of high-dose corticosteroids and mycophenolate, leading to gradual improvement over six months. This case highlights the diagnostic overlap between immune checkpoint inhibitor (ICI)-induced scleroderma-like syndrome and EF, emphasizing the need for early recognition and tailored immunosuppression to mitigate immune-related adverse events (irAEs) while balancing cancer therapy. Awareness of atypical presentations (e.g., hand/face involvement in EF) is critical for timely intervention.

## Introduction

Immune checkpoint inhibitors (ICI) like nivolumab have revolutionized cancer therapy [1]. However, they are associated with immune-related adverse events (irAEs), including rheumatologic manifestations like inflammatory arthritis, myositis, Sjogren's, and scleroderma-like syndromes [2]. Scleroderma and eosinophilic fasciitis (EF) are rare irAEs with overlapping features [3]. We report an unusual case of nivolumab-induced fasciitis mimicking scleroderma, highlighting the diagnostic challenges and the importance of recognizing this potential overlap.

## Case presentation

**Figure 1:**
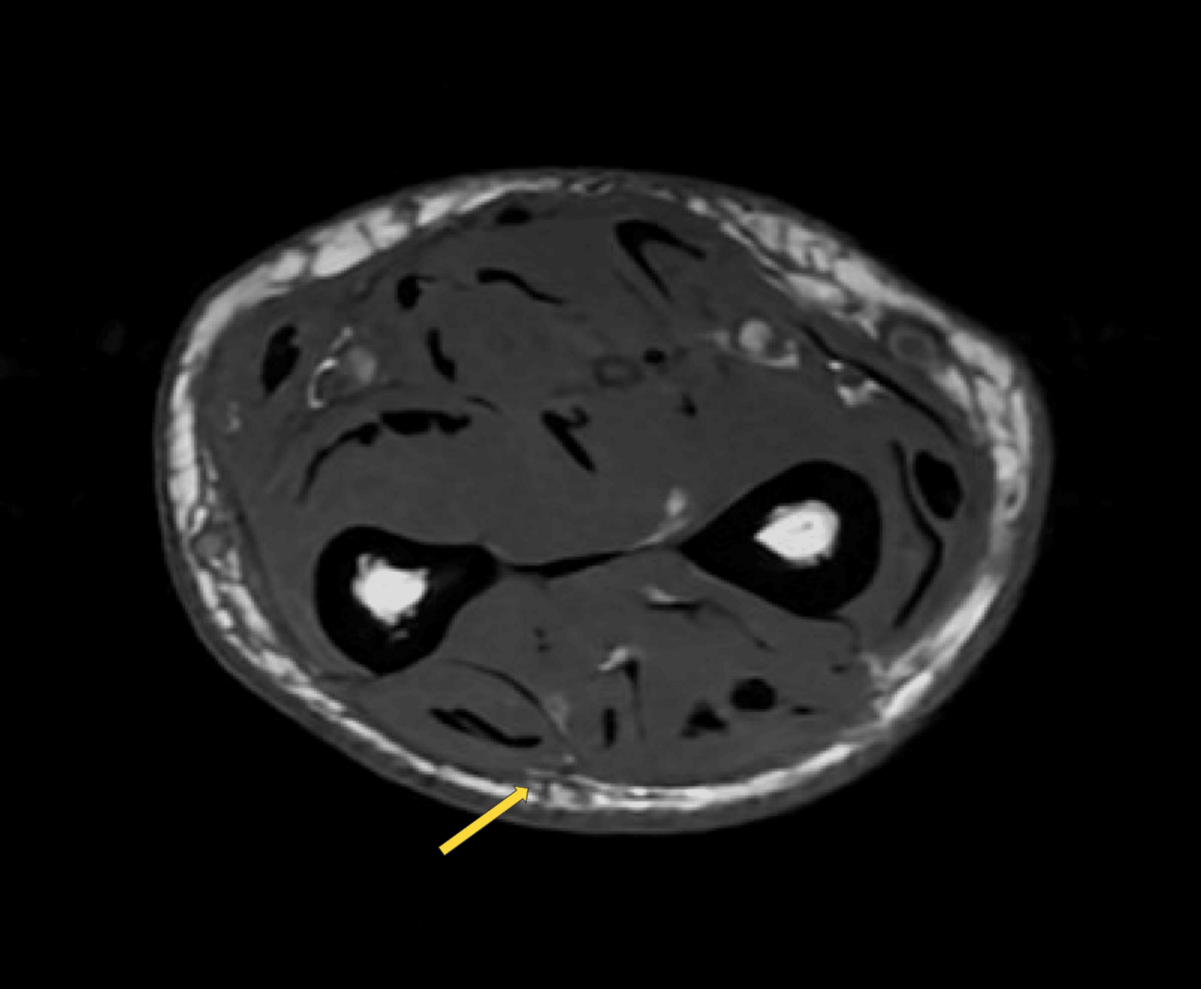
Nonspecific diffuse fasciitis Fascial thickening, increased signal intensity on fluid-sensitive sequence and enhancement after contrast administration on MRI.

A 74-year-old male with desmoplastic melanoma treated with nivolumab developed pain, itching, and swelling in his extremities after five months of treatment. His skin progressively tightened around his mouth and extended down from his elbows and knees distally to his hands and feet. Examination revealed woody induration, a positive prayer sign, and a "sandpaper rash." Due to the skin tightness, the patient had a significantly reduced range of motion of his extremities, jaw tightening, and difficulty swallowing. He also experienced unintentional weight loss, dyspnea, dysphagia, and chronic diarrhea. Notably, his hands and feet also had significant skin thickening and induration. He did not, however, have digital ulcers, Raynaud’s phenomenon, or telangiectasias. Initial serologic studies were positive for antinuclear antibody (ANA) and ribonucleoprotein (RNP) antibodies, as shown in Table 1. The erythrocyte sedimentation rate (ESR) was moderately elevated (40 mm/hr, normal <20 mm/hr), and creatine kinase (CK) levels were normal. Given these findings, systemic sclerosis was initially suspected. The patient was treated with a rapid prednisone taper, mycophenolate, and briefly with tocilizumab (targeting systemic sclerosis while minimizing broad immunosuppression given his active melanoma). However, his symptoms progressed. Scleroderma mimics were considered; Scleroedema was ruled out, given the lack of preferential involvement of the back, neck, and shoulders. Absence of skin papules and paraproteinemia on blood work ruled out Scleromyxedema. Moreover, his lab work now showed new development of peripheral eosinophilia at 1100 (normal 0-500 cells/microliter). Magnetic resonance imaging (MRI) was then ordered, which revealed diffuse fasciitis as shown in Figure 1, leading to a revised diagnosis of eosinophilic fasciitis. Treatment was adjusted to a prolonged prednisone taper starting at 1 mg/kg/day, continued mycophenolate, and discontinuation of nivolumab. Over the following six months, the patient showed gradual improvement in skin thickening, range of motion in his extremities, and normalized eosinophil count, and therefore, mycophenolate was tapered off eventually. He was monitored closely by oncology for signs of recurrence of melanoma off immunotherapy, but he remained in remission.

**Table 1:** Initial serologic studies Anti-RNP: ribonucleoprotein antibody; SSA: Sjögren's syndrome A; SSB: Sjögren's syndrome B.

Serology	Result
ANA	Positive 1:160 homogenous pattern
Anti-RNP	Positive 4.1 (normal <1)
Anticentromere	Negative
Anti-Smith	Negative
SSA	Negative
SSB	Negative
Anti-Scl 70	Negative

## Discussion

ICI therapy leads to programmed cell death protein 1 (PD-1) blockade with overactivation of B and T cells, leading to the release of various cytokines, which promote the activation and recruitment of eosinophils into the fascia, causing inflammation. This inflammation leads to an imbalance in the production of extracellular matrix components, like collagen, resulting in excessive fibrosis, which manifests as thickening and hardening of the skin, particularly in the limbs, a hallmark of EF. Transforming growth factor beta (TGF-β) is a cytokine that plays a crucial role in fibrosis. This case demonstrates the challenges in differentiating between ICI-induced scleroderma-like syndrome and eosinophilic fasciitis [3]. The patient exhibited features of both conditions, including skin tightening, distal extremity involvement, and esophageal dysmotility (suggestive of scleroderma), alongside imaging findings consistent with fasciitis. While EF typically spares the hands, feet, and face, this patient had involvement of these areas, underscoring the spectrum of irAEs associated with ICIs [3]. Early recognition is crucial, as prompt corticosteroid therapy can lead to clinical improvement [4]. EF typically develops weeks to months after nivolumab initiation, and Improvement is usually seen within weeks to months after treatment, which involves prednisone (0.5-1 mg/kg/day) as first-line, with methotrexate or other immunosuppressants, including mycophenolate, rituximab, or intravenous immunoglobulin (IVIG) for refractory cases. Most patients improve with treatment, but symptoms may persist, and nivolumab often needs to be discontinued. Rechallenge carries a risk of recurrence.

## Conclusions

ICI-induced fasciitis can mimic scleroderma, posing diagnostic dilemmas. This case highlights the importance of considering EF in patients on ICIs presenting with skin tightening and atypical features. Increased awareness facilitates timely diagnosis and appropriate management.
